# MRI Evaluation of Resection Margins in Bone Tumour Surgery

**DOI:** 10.1155/2014/967848

**Published:** 2014-05-26

**Authors:** Simon Vandergugten, Sidi Yaya Traore, Olivier Cartiaux, Frédéric Lecouvet, Christine Galant, Pierre-Louis Docquier

**Affiliations:** ^1^Computer Assisted and Robotic Surgery (CARS), Institut de Recherche Expérimentale et Clinique, Université Catholique de Louvain, avenue Mounier 53, 1200 Brussels, Belgium; ^2^Service de Chirurgie Orthopédique et Traumatologique, Cliniques Universitaires Saint-Luc, avenue Hippocrate 10, 1200 Brussels, Belgium; ^3^Département d'Imagerie Médicale, Cliniques Universitaires Saint-Luc, 10 avenue Hippocrate, 1200 Brussels, Belgium; ^4^Département de Pathologie, Cliniques Universitaires Saint-Luc, 10 avenue Hippocrate, 1200 Brussels, Belgium

## Abstract

In 12 patients operated on for bone sarcoma resection, a postoperative magnetic resonance imaging of the resection specimens was obtained in order to assess the surgical margins. Margins were classified according to MRI in R0, R1, and R2 by three independent observers: a radiologist and two orthopaedic surgeons. Final margin evaluation (R0, R1, and R2) was assessed by a confirmed pathologist. Agreement for margin evaluation between the pathologist and the radiologist was perfect (*κ* = 1). Agreement between the pathologist and an experienced orthopaedic surgeon was very good while it was fair between the pathologist and a junior orthopaedic surgeon. MRI should be considered as a tool to give quick information about the adequacy of margins and to help the pathologist to focus on doubtful areas and to spare time in specimen analysis. But it may not replace the pathological evaluation that gives additional information about tumor necrosis. This study shows that MRI extemporaneous analysis of a resection specimen may be efficient in bone tumor oncologic surgery, if made by an experienced radiologist with perfect agreement with the pathologist.

## 1. Introduction

Complete surgical resection remains the gold standard management for bone and soft tissue sarcomas [1, 2]. During the procedure, the surgeon removes the tumour* en bloc*, with a continuous surrounding layer of healthy tissue as the “safe” margin. As most of these tumours are very close to major nerves or vessels, a meticulous preoperative planning is essential in order to spare these structures. Surgeons currently rely on tumour delineation by magnetic resonance imaging (MRI) to plan the surgery.

Postoperatively, the pathologist assesses the achieved surgical margins through rigorous microscopic evaluation [3]. The goal of surgery is to leave a margin of at least 1 mm of normal tissue around the tumor (R0 resection). Whenever there is some residual tumorous tissue (microscopically R1 or macroscopically R2), revision surgery or postoperative radiotherapy is generally recommended. For some authors, the local and general prognosis are clearly linked to the capacity of the surgeon to perform a R0 resection [4, 5], while for other limb salvage surgery has a higher local recurrence rate but equivalent overall survival relative to amputation. Pathological examination of bone sarcomas is technically very difficult and is time consuming in case of large sarcomas. The purpose of this study was to compare margin evaluation by the pathologist and by MRI to evaluate efficiency and accuracy of margin MRI evaluation. According to the literature review, this concept has not been reported to date in either experimental or clinical settings.

## 2. Materials and Methods

### 2.1. Patient Series

Specimens were obtained from 12 patients, with a median age of 16.5 years (9 to 65 years), operated on for bone sarcoma resection between January 2010 and November 2012 (Table 1). Inclusion criteria were primary bony sarcoma accessible to resection surgery and reconstruction. Exclusion criteria were bone metastases or primary bony sarcoma nonaccessible to the surgery and treated with radiotherapy alone. There were 9 osteosarcomas, 1 chondrosarcoma, 1 Ewing sarcoma, and 1 bone leiomyosarcoma. One of the osteosarcomas was a local recurrence from a periosteal osteosarcoma after cortical resection. The sarcoma was localized in the pelvis for 4 patients (1 iliac bone and 3 sacroiliac joint), in the femur for 3 cases, in the tibia for 4 cases, and in the humerus for 1 case. One patient was already metastatic at the time of the surgery. Surgery was not curative but its indication was to allow the patient to walk. The time between surgery and last follow-up was 2.2 ± 0.9 years (mean ± SD).

### 2.2. Magnetic Resonance Imaging Evaluation

Preoperative MRI of the bone tumor had been obtained in all cases in the month prior to surgery, on average 11 days (1–28 days) before surgery. Axial T1- and T2-weighted images, coronal proton density weighted images with fat saturation, and axial and sagittal T1-weighted images with gadolinium enhancement were obtained.

**Figure 1:**
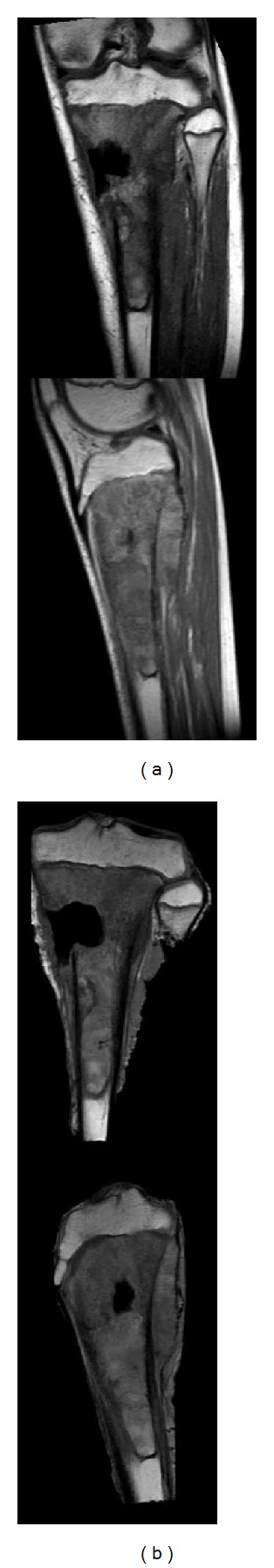
11-year-old patient (n. 5) with osteosarcoma of the proximal tibia. A resection of the tumor was performed with epiphysis resection. The margin was classified R0 by pathologist, radiologist, and senior orthopaedic surgeon. On the left: preoperative MRI. On the right: postoperative MRI of the resected specimen.

**Figure 2:**
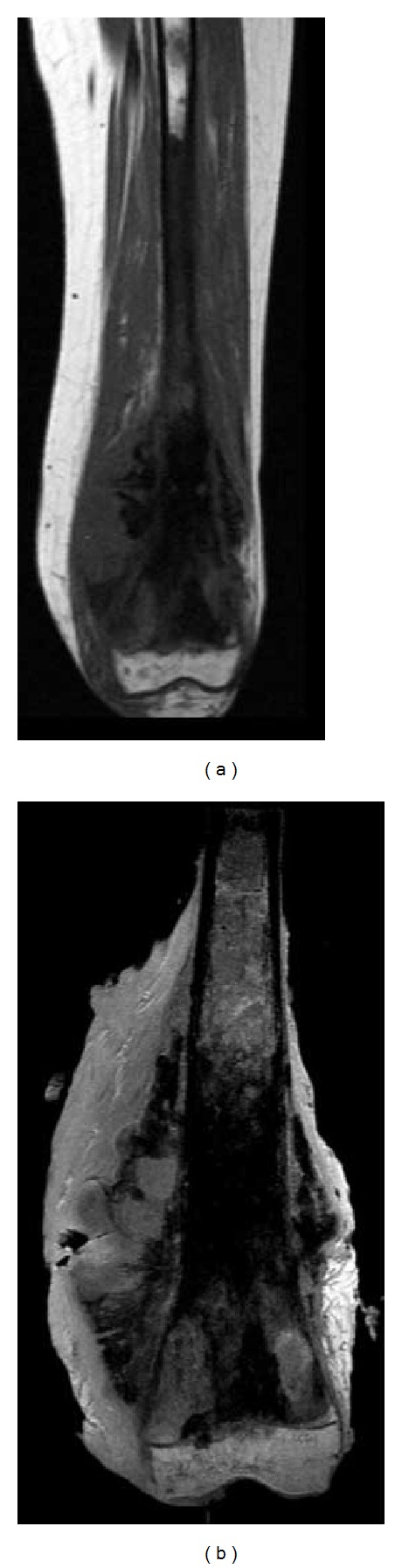
14-year-old patient (n. 7) with osteosarcoma of the distal femur with multiple bone metastases. The tumour was very voluminous and painful and the patient was not able to walk. The goal of the surgery was to allow the child to walk but was not curative. The resection margins were classified R2 by the pathologist and all the observers. On the left: preoperative MRI. On the right: postoperative MRI of the resected specimen.

A postoperative axial 3D-T1-weighted and 3D-T2-weighted MRI of the specimen were performed after resection of the bone tumor. The specimens were oriented according to the long axis of the bone (femur, tibia). For pelvic specimens, the specimen was oriented with scout view to obtain the same slicing compared to the preoperative imaging. The MRI acquisition parameters used for the specimens were specified as follows: reconstruction matrix 176 × 176, section thickness 0.5 mm, and spacing between slices 0.5 mm. The specimens' sequences were saved in DICOM format prior to analysis with the picture archiving and communication system (PACS, Carestream Health, NY, USA). MRI of resected specimens was made postoperatively in the hour following the specimen resection in all but one patient due to MRI unavailability. Specimen MRI was blindly analyzed and classified by three independent observers according to the standardized classification, created by the Union for International Cancer Control (UICC) [6]. They had access to the preresection imaging to help analyzing the specimen MRI. The three observers were an experienced radiologist used to tumor imaging, an experienced orthopaedic surgeon used to tumor surgery, and an unexperienced junior orthopaedic surgeon without experience with bone tumor surgery. UICC classification distinguishes R0 as* in sano* resection with adequate safe margins (Figure 1), R1 as possible microscopic residuals (margin between 0 and 1 mm), and R2 as macroscopic residual disease (Figure 2) [6].

### 2.3. Pathological Evaluation

The method of determining surgical margins is now fairly standardized [3, 7]. The surface of the freshly excised tumour was inked and the specimen was fixed with formalin 4%. Macroscopic serial sections were performed. A first evaluation of the tumour margin was noticed macroscopically and tissues were taken for histology with a special attention for the foci of the closest's margins. These tissue blocs were embedded in paraffin and cut at 5 microns thick before staining with Hematoxylin Eosin. The pathologist analysed then histologically the specimen and measured the distance between the tumour edge and the closest inked surface microscopically. The distance was given in mm. The pathologist was blinded about the result of MRI classification and categorized the margins according to the standardized classification, created by the Union for International Cancer Control (UICC) [6]. The pathologist was not informed of the MRI evaluation results.

### 2.4. Statistical Analysis

The margin classification by the pathologist was considered as the golden standard and was compared with the margin classification obtained by MRI (by the three independent observers) (Table 2). The data were analysed with SPSS software 20.0 (IBM, USA). Kappa method was used to evaluate the agreement between the observers (interobserver). Strength of agreement was evaluated by the kappa value: agreement is perfect if *κ* = 1, very good with *κ* between 0.8 and 1, good with *κ* between 0.6 and 0.8, moderate with *κ* between 0.4 and 0.6, fair with *κ* between 0.2 and 0.4, and poor with *κ* less than 0.2 [8].

## 3. Results

Results of the margin classification by the pathologist and by imaging are listed in Table 2.

Three patients had R2 resection margin at the pathological evaluation. Adjuvant radiotherapy was performed in these patients. Two of them were deceased at the latest follow-up while the third had a good response to chemotherapy and is still alive without evidence of disease.

Agreement for margin evaluation between the pathologist and the radiologist was perfect (*κ* = 1). Agreement between pathologist and experienced orthopaedic surgeon was very good while it was fair between pathologist and junior orthopaedic surgeon (Table 3).

Agreement between the radiologist and the experienced orthopaedic surgeon was very good while it was fair between the unexperienced surgeon and both the experienced surgeon and the radiologist (Table 4).

## 4. Discussion

**Figure 3:**
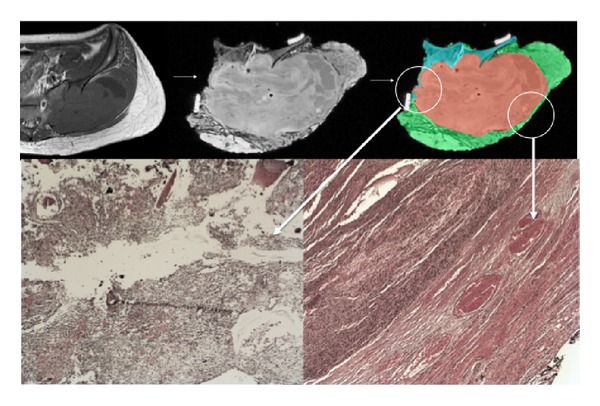
43-year-old patient with undifferentiated osteosarcoma of the sacroiliac joint. Resection was performed with resection of iliac wing and sacral ala. Top-left: preoperative T2-weighted MRI of the tumor. Top-middle: postoperative T2-weighted MRI of the resected specimen. Top-right: segmentation of the tumour has been made on MRI in red color and of the whole specimen in green color for soft tissues and in grey color for bone tissue. Bottom-left: pathological picture of the margin in the encircled area. Tumoral cells are present at the margin, what was already visible on MRI. Bottom-right: a good margin is present at that location.

The goal of this study was to explore the feasibility of imaging resected specimens to give information about the resection margins, to help the fastidious pathological analysis of the specimen. The adjuvant treatment of bone sarcoma is depending on two pieces of information from the pathological analysis of the resected specimen: adequacy of margins and percentage of tumor necrosis. MRI may not give information about the percentage of tumor necrosis; however, MRI image quality was shown to be high enough to give useful information about the resection margins. MRI may not replace pathological analysis but may give information to the pathologist who may focus his analysis on some doubtful areas in case of voluminous specimen (Figure 3).

MRI evaluation of the margin in a freshly resected tumoral specimen seems to be efficient if performed by an experienced radiologist (used to analyze oncologic MRI) or an experienced orthopaedic surgeon (used to oncologic sarcoma surgery). There was a perfect agreement between the pathologist and radiologist and a very good agreement between the pathologist and experienced orthopaedic surgeon. On the contrary, MRI interpretation by the inexperienced surgeon is not recommended, as the agreement is fair.

MRI could be more rapid than histopathology to give a R classification as MRI could be obtained quickly after tumor resection (it takes 45 minutes) and final pathological evaluation is generally not obtained before 1 or 2 weeks in case of bone sarcomas. Nevertheless, MRI may not replace pathologic evaluation of the specimen, as the pathologist will give the final evaluation of the margins and information about the percentage of tumor necrosis.

A theoretical benefit of this procedure could be a direct peroperative margin evaluation by rapid MRI acquisition of the freshly resected specimen. If the MRI is available and if the surgical reconstruction procedure following resection takes several hours, a rapid assessment of the resection margin could be obtained before the end of the surgical procedure, allowing the surgeon to do recut in the region where the MRI assesses an unsafe margin. It would be even possible for the radiologist to come to the operating theatre to show and explain his analysis of the MRI to the surgeon. However, this is not always possible in clinical practice.

The residual tumour classification or R classification may be used after surgical treatment alone, after radiotherapy alone, after chemotherapy alone, or after multimodal therapy [6]. It was described for histologic examination of the margin but after nonsurgical treatment; the presence or absence of residual tumor is determined using methods such as radiologic imaging and biopsy [6]. The R0 (“no residual rumor”) category applies only to cases in which residual tumor cannot be detected by conventional diagnostic methods. A more exact definition should be “no detectable residual tumor.” The R1 category is reserved exclusively for cases in which residual tumor is found by histologic examination. This category may apply to biopsy sampling of the regional tissue at the site of resection or of a distant site at the time of surgery. It also applies to microscopic examination of the resection margins of the surgical resection specimen by the pathologist. R2 applies to cases with macroscopically visible residual tumor that is detected either clinically or pathologically. Despite the fact that R1 has been described for histologic examination, an attempt to use the R classification with MRI has been published [9].

This study has some limitations. All the histologic classification analyses were performed by one observer and one time. Only twelve patients were included in the study. Other experimental studies on animals should show quantitatively the efficiency of margin evaluation by MRI.

## 5. Conclusion

MRI evaluation may not replace the pathological evaluation as it remains the gold standard and gives information about tumor necrosis. Nevertheless, it should be considered as a tool to give quick information about the adequacy of margins and a tool to help the pathologist to focus on doubtful areas. This could help the pathologist to spare time in specimen analysis.

This study shows that extemporaneous analysis of a resection specimen may be efficient in bone tumor oncologic surgery, if made by an experienced radiologist with perfect agreement with the pathologist.

## Figures and Tables

**Table 1:** Patient's data.

*N *	Age (years)*	Gender	Sarcoma location	Sarcoma type	Presence of metastases*	Diameter of the lesion (cm)	Soft tissue mass
1	15	M	Proximal humerus	Osteosarcoma	No	8 × 4.7	Absent
2	63	M	Iliac bone	Chondrosarcoma	No	13.4 × 7.8	Present
3	43	F	Sacroiliac joint	Osteosarcoma	No	6.5 × 12.2	Present
4	12	M	Proximal tibia	Ewing sarcoma	No	8 × 2.4	Absent
5	11	M	Proximal tibia	Osteosarcoma	No	10.6 × 4.6	Present
6	18	M	Proximal tibia	Osteosarcoma (recurrence)	No	5.8 × 2.1	Absent
7	14	F	Distal femur	Osteosarcoma	Multiple bone metastases	20.7 × 7.7	Present
8	9	M	Proximal tibia	Osteosarcoma	No	12.4 × 4.5	Present
9	19	F	Distal femur	Osteosarcoma	No	6.3 × 6.9	Present
10	65	M	Sacroiliac joint	Leiomyosarcoma	No	9 × 6.4	Present
11	15	M	Diaphyseal femur	Osteosarcoma	No	20.7 × 7.6	Present
12	43	F	Sacroiliac joint	Radio induced osteosarcoma	No	10.3 × 6	Present

M: male, F: female.

*At the time of surgery.

**Table 2:** Comparison of pathological evaluation and MRI evaluation.

*N *	Histopathological evaluation		MRI evaluation			Follow-Up (years)	Evolution
	Pathologist	Response to chemotherapy	Radiologist	Experienced orthopaedic surgeon	Unexperienced surgeon		
1	R1	Bad	R1	R1	R1	2.9	NED
2	R1	No chemotherapy	R1	R1	R1	2.7	NED
3	R2	Bad	R2	R2	R2	1.6	Deceased
4	R0	Good	R0	R0	R0	2.9	NED
5	R0	Good	R0	R0	R1	3.1	NED
6	R0	Good	R0	R0	R0	2.8	NED
7	R2	Bad	R2	R2	R2	0.6	Deceased
8	R1	Good	R1	R0	R0	1.9	NED
9	R1	Good	R1	R1	R0	1.7	NED
10	R2	Good	R2	R2	R1	3.6	NED
11	R1	Bad	R1	R1	R0	1.1	NED
12	R1	Good	R1	R1	R2	1.2	NED

R0: *in sano* resection with adequate safe margins (margin > 1 mm); R1: possible microscopic residuals (margin between 0 and 1 mm); R2: macroscopic residual disease; NED: no evidence of disease.

**Table 3:** Agreement between pathological evaluation and MRI evaluation.

Agreement with the pathological evaluation	Kappa value	*P* value
Radiologist	*K* = 1 (perfect agreement)	<0.0001
Experienced orthopaedic surgeon	*K* = 0.87 (very good agreement)	<0.0001
Unexperienced orthopaedic surgeon	*K* = 0.25 (fair agreement)	0.2

**Table 4:** Interobserver agreement observers with MRI evaluation.

Agreement	Experienced orthopaedic surgeon
Radiologist and experienced orthopaedic surgeon	*K* = 0.71 (*P* value < 0.0001)
Radiologist and unexperienced orthopaedic surgeon	*K* = 0.25 (*P* value = 0.02)
Experienced orthopaedic surgeon and unexperienced surgeon	*K* = 0.37 (*P* value = 0.07)

## References

[1] Lewis VO (2009). What’s new in musculoskeletal oncology. *Journal of Bone and Joint Surgery A*.

[2] Docquier P, Paul L, Cartiaux O, Delloye C, Banse X (2010). Computer-assisted resection and reconstruction of pelvic tumor sarcoma. *Sarcoma*.

[3] Rubin BP, Fletcher CDM, Inwards C (2006). Protocol for the examination of specimens from patients with soft tissue tumors of intermediate malignant potential, malignant soft tissue tumors, and benign/locally aggressive and malignant bone tumors. *Archives of Pathology and Laboratory Medicine*.

[4] Stoeckle E, Gardet H, Coindre J-M (2006). Prospective evaluation of quality of surgery in soft tissue sarcoma. *European Journal of Surgical Oncology*.

[5] Zagars GK, Ballo MT, Pisters PWT, Pollock RE, Patel SR, Benjamin RS (2003). Surgical margins and reresection in the management of patients with soft tissue sarcoma using conservative surgery and radiation therapy. *Cancer*.

[6] Wittekind C, Compton CC, Greene FL, Sobin LH (2002). TNM residual tumor classification revisited. *Cancer*.

[7] Abdul-Karim FW, Bauer TW, Kilpatrick SE, Raymond KA, Siegal GP (2004). Recommendations for the reporting of bone tumors. *Human Pathology*.

[8] Altman DG (1991). *Practical Statistics for Medical Research*.

[9] Bellanova L, Schubert T, Cartiaux O (2014). MRI-based assessment of safe margins in tumor surgery. *Sarcoma*.

